# “From the river to the sea, Palestine will be free” – a chant that hampers the very foundation of malaria elimination around the world

**DOI:** 10.5281/zenodo.15740720

**Published:** 2025-06-25

**Authors:** Anton Alexander

**Affiliations:** 1 BC Business Centrum, Elscot House, Arcadia Avenue, London N3 2JU, United Kingdom. 2 Editor, MalariaWorld Journal.

Since 7th October 2023, when Hamas from Gaza massacred Jews in Israel, a chant “From the river to the sea, Palestine will be free” has become fashionable and may be seen on banners and heard as chants in many anti-Israel marches around the world. The consequence, certainly since 7th October, has been that anything associated with Zionism or Israel is vilified, boycotted or shunned in certain quarters.

Thus, by choosing to boycott anything that had a connection with Zionism or Israel, a study of the steps and events leading to the launch of the first start of sustainable malaria control has been missed, discouraging the malaria community of an opportunity to consider why and how certain methods were employed to achieve a desired successful outcome. There appears to be an attempt to suppress the fact that Palestine over 100 years ago was desolate, it was either almost empty or uninhabitable in many areas, due to malaria. In 1919, Dr Manson-Bahr, a future director of the London School of Hygiene and Tropical Medicine, described Palestine as one of the most highly malarious countries in the world.

During WW1, Turkey allied itself with Germany against England. In 1918, in the final battle in the Middle-East in WW1, the British Army, having taken steps to protect itself and temporarily control the malaria whilst in southern Palestine, attacked the Turkish Army and advanced from the British ‘healthy’ line into the ‘unprotected’ northern half of Palestine on 19th September 1918. It was then known that the incubation period of malaria after a bite by an infected mosquito was generally 10 days. During the following 10 days after 19th September - in fact during the incubation period - the Turkish Army was decisively defeated. However, fortunately for the British Army and only after the Turkish defeat, from 1st October onwards, over half the British Army, over 20,000 British troops, was to either collapse or ultimately die from malaria due to exposure to the bite of an infected mosquito sustained whilst behind the unprotected Turkish lines during that 10 day time-frame. Of the Turkish prisoners taken after the defeat, over 20,000 had to be immediately hospitalised with malaria.

The method to **sustainably** control malaria, the first successful attempt anywhere in the world to do so, was developed by the Zionists in Palestine in 1922. It was born out of necessity, otherwise any attempt by the Jews to settle in Palestine would not have been possible. Land occupied by the Jews would have been usually vacant because of the severity of the disease, often deemed uninhabitable, but being the only land ever offered to them for purchase. Such land was only rendered habitable eventually by the Zionist anti-malaria works and effort. Malaria was declared eliminated in Israel by the WHO in 1967.

A misleading description of Palestine 100 years ago, often by those hostile to the very existence of the State of Israel, is of a country densely populated by Arabs. It is perhaps an attempt to suggest malaria never existed in Palestine, or at best, it was never severe. If the suggestion was correct, if there was no malaria, then the subject of malaria control/ elimination would not be relevant. It is as if the world has somehow, somewhere, sometime lost touch with history and scientific records and evidence. There appears a depressing similarity in this attempted suppression of facts with Germany in the 1930s when, because any association with Jews was to be avoided, even the music of Mendelssohn, a Jew, was banned by the Nazis!

Perhaps there was always a tendency within the malaria community not to refer to the Palestine malaria elimination method and the following could be the explanation. The WHO Handbook for Integrated Vector Management [1] helpfully included a brief historical note about malaria elimination. It states:

*“Before the Second World War, vector control was conducted predominantly by environmental control of the proliferation of mosquitoes. [e.g., the 1922 Palestine method] …. There is evidence that environmental management had a clear impact on disease; however, elimination of disease was never on the agenda. The advent of DDT and other organochlorine pesticides during the 1940s changed this situation. … The focus of vector control on insecticides meant that environmental management and other alternative methods were **underexploited or even forgotten**.”*

But now, in particular since 7th October, it has become extremely ‘unfashionable’ to suggest Palestine 100 years ago was desolate, almost empty. Such desolation would contradict the chant ‘From the river to the sea, Palestine will be free’ with its erroneous suggestion the country 100 years ago was densely inhabited by Arabs who were subsequently displaced by the Jewish Zionists.

It is now accepted within the malaria-community that progress in the fight against malaria has dangerously stalled in recent years, highlighting the importance of new interventions and tailored approaches. A critical factor that must be considered across contexts and interventions is human behaviour. Whilst it may be considered ‘unfashionable’ to some, it is now essential and urgent that the sustainable method of malaria-control successfully developed and employed in Palestine from 1922 onwards be widely studied.
